# Moderate Intensity Training Impact on the Inflammatory Status and Glycemic Profiles in NOD Mice

**DOI:** 10.1155/2015/737586

**Published:** 2015-08-05

**Authors:** Roberto Codella, Giacomo Lanzoni, Alessia Zoso, Andrea Caumo, Anna Montesano, Ileana M. Terruzzi, Camillo Ricordi, Livio Luzi, Luca Inverardi

**Affiliations:** ^1^Department of Biomedical Sciences for Health, University of Milan, Milan, Italy; ^2^Diabetes Research Institute, University of Miami Miller School of Medicine, Miami, FL, USA; ^3^Metabolism Research Center, IRCCS Policlinico San Donato, San Donato Milanese, Italy; ^4^Division of Metabolic and Cardiovascular Science, Metabolism, Nutrigenomics and Cellular Differentiation Unit, San Raffaele Scientific Institute, Milan, Italy

## Abstract

The nonobese diabetic (NOD) mouse represents a well-established experimental model analogous to human type 1 diabetes mellitus (T1D) as it is characterized by progressive autoimmune destruction of pancreatic *β*-cells. Experiments were designed to investigate the impact of moderate-intensity training on T1D immunomodulation and inflammation. Under a chronic exercise regime, NOD mice were trained on a treadmill for 12 weeks (12 m/min for 30 min, 5 d/wk) while age-matched, control animals were left untrained. Prior to and upon completion of the training period, fed plasma glucose and immunological soluble factors were monitored. Both groups showed deteriorated glycemic profiles throughout the study although trained mice tended to be more compensated than controls after 10 weeks of training. An exercise-induced weight loss was detected in the trained mice with respect to the controls from week 6. After 12 weeks, IL-6 and MIP-1*β* were decreased in the trained animals compared to their baseline values and versus controls, although not significantly. Morphometric analysis of pancreata revealed the presence of larger infiltrates along with decreased *α*-cells areas in the control mice compared to trained mice. Exercise may exert positive immunomodulation of systemic functions with respect to both T1D and inflammation, but only in a stringent therapeutic window.

## 1. Introduction

Type 1 diabetes (T1D) is an autoimmune disease in which the immune system is activated against the insulin producing *β*-cells present in the pancreatic islets [1]. The elimination of these cells impairs the mechanisms that control the glucose blood level and, up to now, the possible and most efficacious treatment for T1D is based on insulin administration and pancreas or pancreatic islet transplantation [2, 3]. While exercise represents a cornerstone in the treatment and prevention of type 2 diabetes mellitus, only recently its effects on glycemic profile and autoimmunity in T1D have been debated by several studies, due to the controversial data reported regarding the benefits of supervised exercise training on metabolic control in people with T1D [4, 5]. In fact, managing blood glucose in T1D while exercising is complex and involves multiple factors such as type of insulin used, time between food intake and sport activity, duration, and intensity of physical effort, known to deter people from enjoying the benefits of sport at both recreational and competitive levels [6, 7]. Some speculative hypotheses instead point at the positive immunomodulatory effect of exercise with respect to both T1D and inflammation [8, 9]. Orešič et al. showed how metabolic dysregulation precedes the autoimmune attack against *β*-cells and suggested lifestyles modification as front-line prevention strategy [10]. During exercise, skeletal muscle releases myokines (IL-6) and the subsequent production of IL-1 receptor antagonist (IL-1ra) by monocytes and macrophages may frame a potent anti-inflammatory window [11].

There are several studies investigating the effects of exercise in inbred rodents with naturally occurring type 2 diabetes or streptozotocin-induced diabetes [12, 13]; however, few studies have been conducted in a NOD mouse model which spontaneously develops T1D [14–17] and resembles the disease progression in humans. By using the NOD model, we tested the effect of moderate-intensity exercise in inducing immunological changes that would result in the delay of T1D progression. In particular, we analyzed the differences in cytokine profiles between trained and untrained animals and correlated these modulations with T1D appearance.

## 2. Material and Methods

### 2.1. Animals and Training

Eight-week-old female NOD mice (Jackson Laboratory) were first acclimated to a six-lane treadmill (Columbus, OH) by running for 10 min at 10 m/min followed by 2 min at 20 m/min (0° slope), in three running sessions over a week before exercise testing. Animals were then trained on a treadmill following a chronic exercise regime (30 min/day, 5 days/week at the speed of 12 m/min) for 12 weeks. Age-matched, control animals were left untrained. Prior to and upon completion of the training period, mice underwent muscular performance evaluation determined by a submaximal incremental test (peak oxygen uptake (VO_2_) challenge), as previously described [16]. To determine VO_2_ peak, mice were placed in a treadmill for 5 min at a 0° incline and 0 m/min. The NOD mice were then challenged with 1.5 min intervals of increasing speed at a 15° incline. Nonfasting glycemic levels of experimental mice were monitored 2-3 times a week using OneTouchUltra2 glucometers (Lifescan). Animals were considered diabetic and euthanized upon 3 consecutive blood glucose readings above 300 mg/dL. Animal studies were performed in compliance with IACUC and the US Department of Health and Human Services Guide for the Care and Use of Laboratory Animals.

### 2.2. Cytokines Bead Assay

Cytokine profiles were evaluated on the serum of the NOD mice before and after 8 weeks of exercise training and upon completion of the 12-week endurance training program. The serum content of G-CSF, GM-CSF, IFN-*γ*, IL-1*β*, IL-2, IL-4, IL-6, IL-10, IL-2p40, IL-13, MCP-1, MIP-1*β*, MIP-2, VEGF, and TNF-*α* was analyzed simultaneously using the cytokine bead assay (Millipore) and data were collected using the Luminex.

### 2.3. Immunohistochemistry and Images Acquisition

Pancreas of 20-week-old NOD mice was collected and fixed in buffered formalin 10% overnight at 4°C and subsequently in optimal cutting temperature (OCT, Tissue-Tek) compound and frozen in dry ice. 5 *μ*m sections were obtained from the cryopreserved blocks, permeabilized with 0.3% PBS Triton X-100, then incubated in universal blocker reagent (Biogenex San Raman CA) for 30 min, and washed in Optimax wash buffer (Biogenex). Sections were then incubated overnight at 4°C with guinea pig anti-insulin (Biogenex ready to use polyclonal antibodies) and mouse antiglucagon (monoclonal clone K79bB10 diluted at 1 : 1000, Sigma) primary antibodies and for 1 hr at room temperature with goat anti-guinea pig Alexa Fluor 488 (Invitrogen) and goat anti-mouse Alexa Fluor 568 (Invitrogen) secondary antibodies diluted in wash buffer at 1 : 300. As negative control samples, slides were incubated with the preimmune serum from animal of the same species instead of the primary antibody. DAPI (Invitrogen) was used for nuclear staining and slides were mounted with ProLong Antifade (Invitrogen). Pictures were acquired with a Zeiss Axiovert 200 M confocal microscope, accessorized with a Hamamatsu camera, available at the imaging core facility of the Diabetes Research Institute, University of Miami. Slides analyzed at the University of Milan were acquired with Nikon Eclipse 50I microscopy and images were captured using Nis-Elements D 4.00 software (Nikon Instruments Europe BV, Netherlands). Data were displayed and analyzed using Adobe Photoshop CS4. A Scale bar 200 *μ*m or (20x) was used.

### 2.4. Automated Quantification

Quantification of cellular composition (i.e., *β*-cells, *α*-cells, and nuclei) was performed by using the cells count macrowritten for Image J (http://imagej.nih.gov/ij/).

### 2.5. Statistical Analysis

Glucose profiles were shown as median and quartiles given the nonparametric distribution of the data, except where differently expressed. Muscular performance data were presented as the mean ± SEM and *t*-tests were used to calculate statistical significancy. Cytokines were analyzed by a two-way ANOVA with repeated measures followed by Bonferroni's post hoc test. Since the distributions of G-CSF, GM-CSF, and IL-10 showed deviations from normality due to the presence of outliers, these cytokines were subjected to logarithmic transformation prior to the execution of ANOVA. Results were considered significant when *p* ≤ 0.05.

## 3. Results

### 3.1. Glucose Profiles

**Figure 1:**
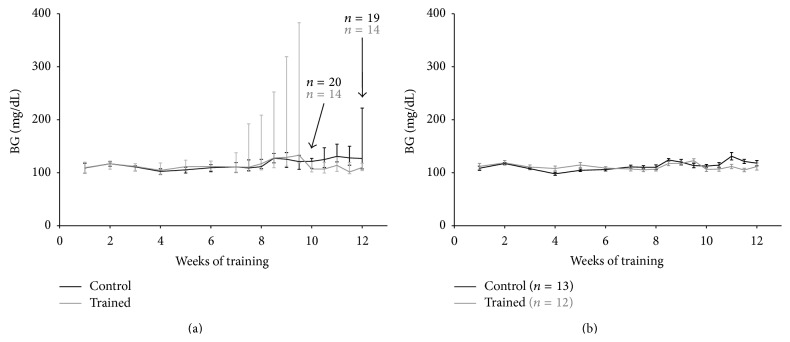
Glycemic profiles over the 12-week exercise training program in all NOD mice (a) and in diabetes-free NOD mice (b). In (a), glycemic values are expressed as median and interquartiles, whereas in (b) data are shown as means and SEM.

**Figure 2:**
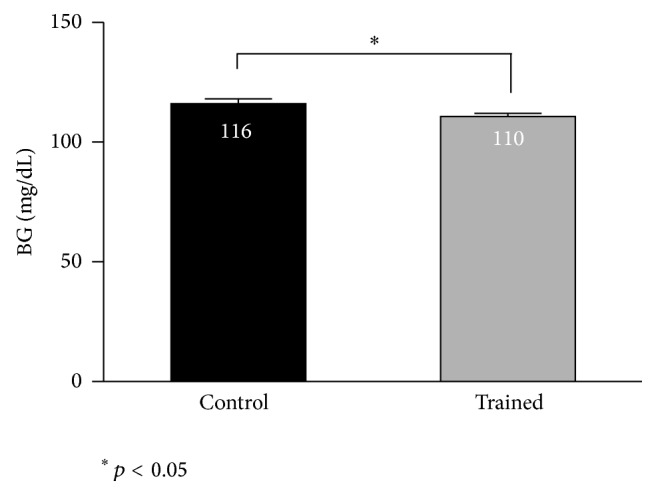
Average glycemic values in diabetes-free NOD mice in the time-window 6–12 weeks of training. Results are shown as means and SEM.

Deterioration of glycemic profile was appreciable around the 10th week of training in all NOD mice under investigation, with slight lower glycemia in the exercising mice with respect to the untrained ones (Figure 1(a)), although the impact of diabetes was remarkable already at week 6 in the trained mice, as indicated by the interquartiles and the diabetic incidence. A similar pattern was detectable among the diabetes-free mice (Figure 1(b)), with a better glycemic profile in the NOD mice on training compared to the controls. In the second part of the experiment, from weeks 6 to 12 of training, among the diabetes-free mice, the average glycemic values resulted in being significantly lower in the trained animals with respect to the controls (110 ± 1.4 versus 116 ± 2 mg/dL, means and SEM, *p* < 0.05) (Figure 2).

### 3.2. Body Weight

An exercise-induced weight loss was registered in the trained mice after 6 weeks of training as compared to the sedentary mice (Figure 4(a), *p* < 0.01). From week 7, the exercising mice continued to weigh less than their age-matched controls, and they remained leaner until the end of the experiment (Figure 4(b), *p* < 0.01).

### 3.3. Incidence

**Figure 3:**
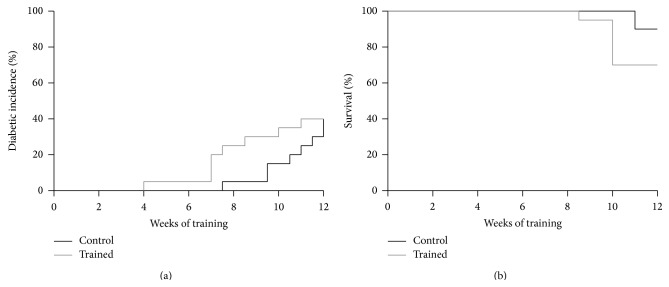
Incidence of type 1 diabetes (a) and survival (b) in all NOD mice.

At training week 7, 4 out of 20 exercising mice became diabetic and that reflected the diabetic incidence percentage, although not significantly, between exercising (10%) and control mice (3%) (Figure 3(a)). After 12 weeks of training, diabetic incidence was 40% for both groups. In detail, 2 out of 14 mice on training were diabetic, whereas, among the controls, 5 out of 18 mice were diabetic. On the other hand, a substantial number of animals remained diabetes-free at the end of the experiment (12 trained mice versus 13 controls) (Figure 2).

### 3.4. Survival

The survival rate declined progressively in both groups, with no statistical difference between the exercising mice (70%) and the controls (88%), upon completion of the 12-week training program. This final rate was indirectly linked to the early diabetic incidence occurring in the trained mice, starting from week 7. In fact, those 4 mice that became diabetic after 7 weeks of training died concurrently during week 10. Overall, 6 mice of the exercising group died because of diabetes; likewise 2 control mice faced the same fate.

### 3.5. Muscular Performance

A submaximal incremental running test was performed at baseline and upon completion of the 12-week chronic training program to determine the acute exercise capacity of the NOD mice, putatively conditioned by the endurance training* per se* and, above all, by the diabetes progression. To evaluate this, we differentiated the results coming from all NOD mice (trained versus controls, regardless of the diabetes diagnosis; Figures 5(a) and 5(c)) and those arisen from the comparison on all diabetes-free NOD mice (trained versus controls, without diabetes; Figures 5(b) and 5(d)). At the end of 12 weeks of training, maximal running speed assessed during the submaximal test was found to be significantly decreased in all NOD mice with respect to their pretraining values (*p* < 0.05), whereas no difference was found between trained and control animals when comparing their posttraining values (Figure 5(a)). When diabetes-free mice were compared, trained mice worsened their maximal running speed at 12 weeks with respect to their baseline values (Figure 5(b), *p* < 0.01). Similarly, a significant reduction in the distance covered during the submaximal performance test was also found in the NOD-exercising mice at week 12 with respect to their pretraining values (Figure 5(c), *p* < 0.01) and the age-matched controls also (Figure 5(c), *p* < 0.05). However, even the control mice deteriorated their performance after 12 weeks as compared to the maximal distance covered at baseline (Figure 5(c), *p* < 0.05). A significant decrease in the maximal distance was also found in the diabetes-free trained mice at week 12 as compared to week 0 (Figure 5(d), *p* < 0.01).

### 3.6. Morphometric Analysis of Pancreata

Diabetes negatively affected the morphology of the islets in both trained and control groups. Typical islet from a diabetic exercising animal after 6 weeks of training is shown (Figure 6(a)) in the pancreatic section triple stained for insulin, glucagon, and nuclei. Immunostaining of pancreatic sections indicated the presence of larger infiltrates in control mice, along with a greater amount of *α*-cells in the NOD-exercising mice at the end of the 12-week training program (Figures 6(b), 6(c), and 6(f); *p* = 0.052). Pancreatic infiltrates were ubiquitously detected in mice on training and controls, whereas no *β*-cells were found at the end of the study in all diabetic mice.

### 3.7. Cytokines

Serum pro- and anti-inflammatory cytokines measured at the baseline, after 8 weeks of training and at the conclusion of the 12-week training program, fluctuated with no statistical significance throughout the timeline in all NOD mice (Figure 7). Only some cytokines seemed to be aligned to an exercise-promoted anti-inflammatory pattern: IL-6 and MIP-1*β* resulted in being lower, even though not significantly, at the termination of the training program in the trained animals compared to the sedentary controls, and with respect to their pretraining values.

## 4. Discussion

With this in vivo approach, we wanted to evaluate how physical activity as endurance training can contribute in controlling T1D progression and analyzing the role played by exercise on the immune system. Preliminary studies have shown the importance and impact of physical exercise on controlling autoimmune diseases, by modulating different immune parameters and components [11, 18–23]. Most studies on the effects of exercise on immunity have shown that habitual, moderate-intensity training augments immune responses by increasing the expression of proinflammatory cytokines, including IL-2, IL-1*β*, and TNF-*α*, while exhaustive exercise tends to be immunosuppressive [24–26]. On the basis of our data, NOD mice appeared to be interesting for the studying of insulin-dependent diabetes although they remained a questionable model for running exercise studies with high significance for the autoimmunity [27]. In fact, in NOD mice, diabetogenesis is under complex polygenic control and the penetrance of these polygenes is greatly conditioned by wide disparate conditions (microbial and physical environment, diet, etc.): some environmental pathogens may even offer more protection to NOD mice against the T1D onset, by the simple activation of their immune system [28]. In this perspective, our hypothesis to exploit the positive stimulation of physical exercise was tricky to be challenged. On a side, exercise, under adequate mild doses, would have counteracted the activation of diabetogenic innate immune responses or, at least, it would have exerted those preventive, protective effects in T1D by decreasing oxidative stress and preservation of *β*-cell integrity. However, there is incomplete knowledge on how exercise acts in T1D, and therefore physical activity could be as stressogenic as many other detrimental conditions for which NOD mice are highly susceptible to develop diabetes. In our study, the design failed to demonstrate a delay in the onset of T1D thanks to exercise; on the contrary, an early diabetic incidence occurred in the exercising group (*n* = 4) as early as week 7 of the training program, although not significantly. Cumulative diabetic incidence was though consistent with data of the literature, confirming expected percentages of T1D penetrance in this model [28].

Certainly, diabetes progression affected* per se* the exercise capacity of the mice irrespective of the endurance training: physical exercise represented an additional stress to the NOD-exercising mice, even at moderate intensity. However, those NOD mice, able to tolerate the exercise stress, showed at the same time a slightly ameliorated inflammatory metabolic profile, one that may be contrasting the diabetes progression and the inflammation attack toward the pancreatic islets. Overall, exercise neither was able to modulate and control the immune attack toward autologous *β*-cells even if initiated prior to the T1D onset, nor had ability to rescue *β*-cells once lost, in accordance with previous studies [12, 13, 29]. However, after 10 weeks of training, diabetic and nondiabetic trained mice showed better glucose profiles than the control mice (Figures 1 and 2).

Today the mechanisms by which exercise regulates glycemia in a T1D context are still unclear. Previously, very few studies documented the possibility to positively control and lower blood glucose by means of exercise in T1D animal models [30]. Some of them were designed on rat models, using streptozotocin-induced diabetes, which rules out autoimmunity, a leading feature resembling human T1D [12, 13, 20, 29–31]. NOD-SCID mice were also studied because they do not develop T1D, since they do not have a functional immune system. Other studies relied on exogenous insulin treatments to reduce blood glucose, which provides disadvantages when interpreting data, because high glucose is toxic to the islet cells, and glucotoxicity represents a biasing factor [32, 33]. In the present study, NOD mice were not treated with insulin. Exercise had glucose-lowering effects, reaching statistical significance only in the late window of the observed training period (Figures 1(a) and 2).

The significant reduction of blood glucose was paralleled also in diabetes-free NOD mice, particularly when referring to the mean glycemic values (Figures 1(b) and 2). This could suggest a preserved ability by which exercise downregulates blood glucose, in NOD mice, when the immune attack has not led yet to frank diabetes, that is, by insulin-stimulated glucose uptake in the skeletal muscle [34].

**Figure 4:**
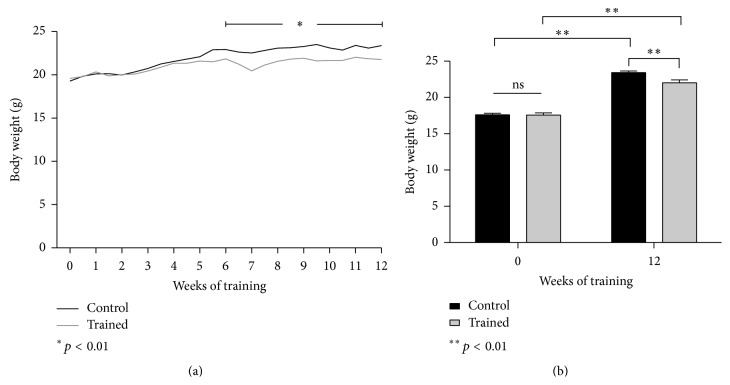
(a) Body weight throughout the 12-week training program. (b) Body weight difference at baseline and upon completion of the 12-week training program. Results are shown as means and SEM.

**Figure 5:**
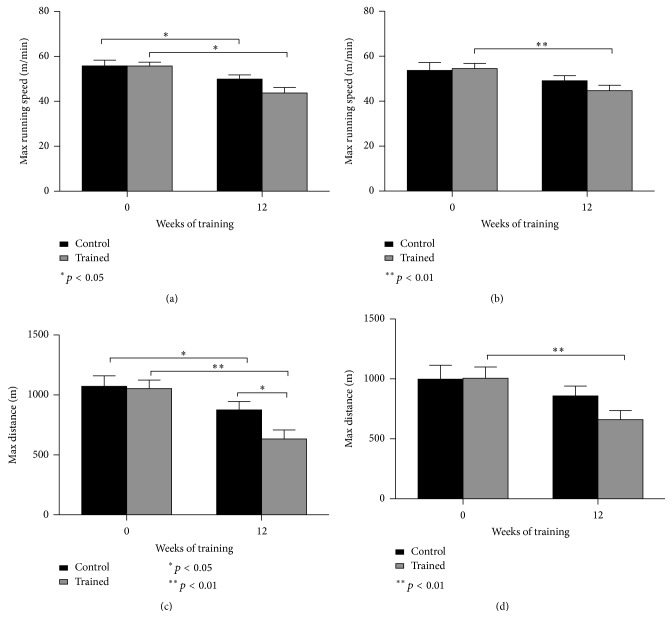
Muscular performance data: speed and distance obtained during a submaximal incremental running test, respectively, in all NOD mice (a, c) and in diabetes-free NOD mice (b, d). Results are shown as means and SEM.

Over the 12-week study, both groups exihbited an expected increase in body weight (+15–25%); however, from week 6, exercising mice began to display significant reductions in body weight compared to controls (−15%) which remained significantly lower (*p* < 0.01) at the termination of the study, despite only a 1.4 g difference (Figure 4). Unfortunately, we were not able to ascertain whether this discrepancy was due to a simple exercise-induced leaner mass rather than a more cachectic state: no visible signs or discomfort or stress was though detected, except for polyuria in the diabetic mice.

A nonunivocal inflammatory pattern emerged from the current analysis. In addition, the expression profiles of serum inflammatory cytokines were not mirrored in the infiltrating immune cells, which are important mediators for *β*-cell demise in the NOD model [35]. Once precised, some of the cytokines measured in the blood at the termination of the study fluctuated in an anti-inflammatory fashion (IL-6, MIP-1*β*) in the trained mice compared to controls, although without reaching statistical significance. Therefore, these indications must be collected thoughtfully, especially considering the lack of statistical confirmation. Besides, other cytokines (IL-2, IL-4, and IL-10) seemed to act in a proinflammatory way at the end of the study in the exercised mice. However, the important anti-inflammatory cytokine IL-10 was found higher in the serum of the trained mice over the remainder of the study (until week 10) compared to controls (Figure 7).

**Figure 6:**
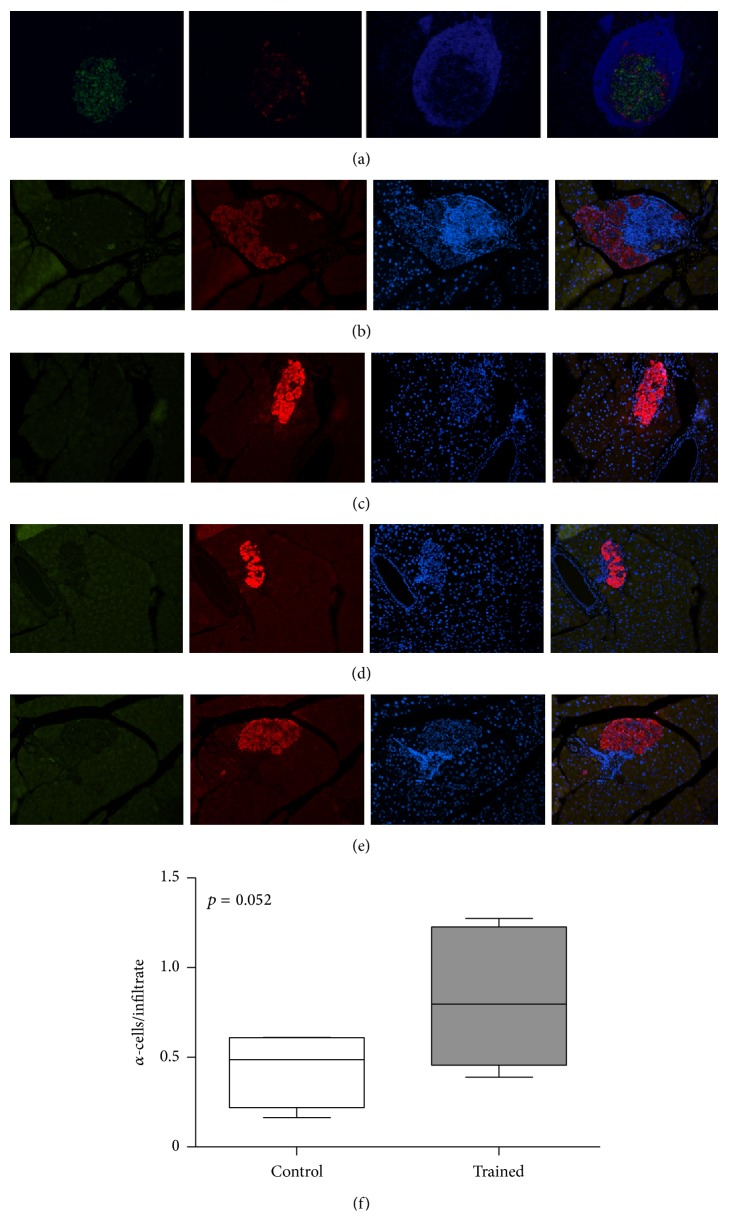
(a) Islet of Langerhans visualized by immunofluorescence microscopy. This figure is a representative immunostaining of pancreatic sections, from a NOD diabetic mouse after 6 weeks of exercise. The entire tissue section is captured by a modified method of “virtual slice image capture” using a microscope with a 10x objective. Each virtual slice taken at multiple fluorescent channels is merged into one composite, shown as insulin (green), glucagon (red), and DAPI for the nuclei. Quantitatively, the cell composition of these mouse islets was more than 70%  *β*-cells and less than 20%  *α*-cells. Analysis of the immune infiltrates (blue) in the pancreatic islets of diabetic untrained (b) or trained (c) animals at 12th week of exercise (20x objective). (d, e) Analysis of the pancreatic islets of two mice died at week 10 of exercise because of earlier diabetic incidence (occurred at 7th week of training) (20x objective). (f) Whiskers plot of the ratio *α*-cells/infiltrate in controls and trained mice at the end of the 12 weeks of training.

**Figure 7:**
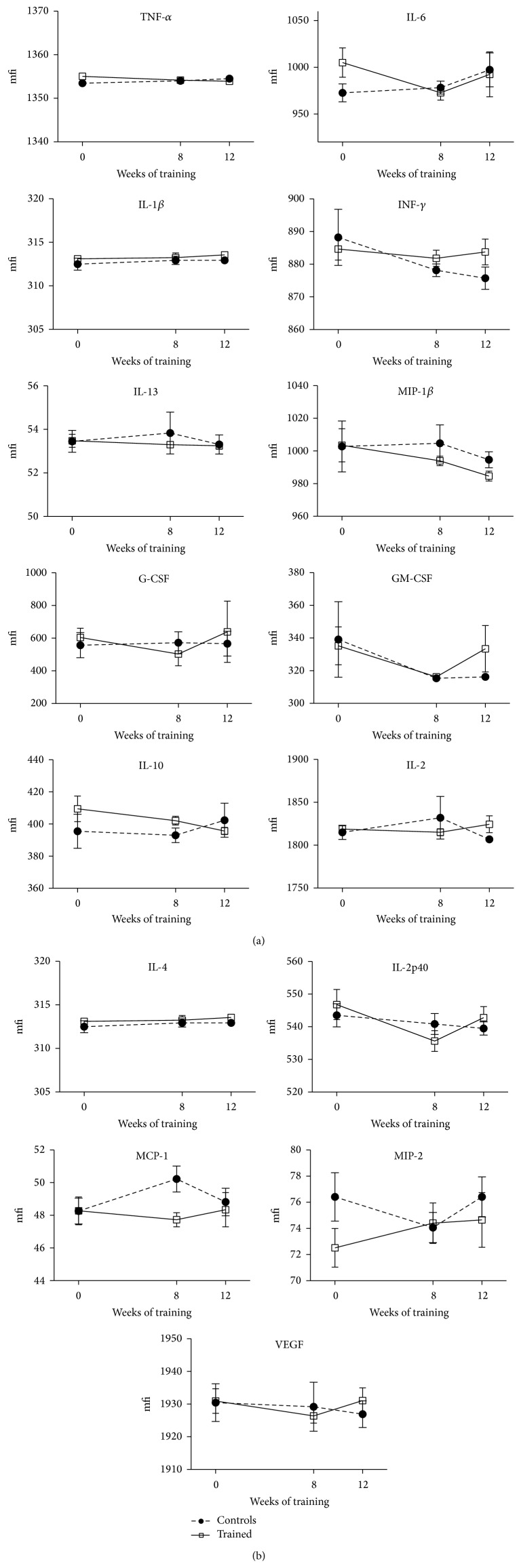
Fluctuations of the cytokines in all NOD mice, throughout the entire duration of the 12-week endurance training program. Results are shown as means and SEM (mfi = mean fluorescence intensity).

As to the inflammatory milieu in NOD mouse islets, the morphological examination indicated a tendency for a decreased estimate of infiltrates in the trained mice along with a greater amount of *α*-cell areas with respect to the diabetic controls (Figure 6(f), *p* = 0.052). Plesner et al. reported the nonbeta islet endocrine cell remodeling in diabetic NOD mice: that study suggested that infiltrating immune cells may restrict alpha-cell expansion in NOD mouse islet in the diabetic state [36]. Here, a greater alpha-cells-infiltrates ratio was also found in the diabetic states of trained mice sacrificed at week 7 of our study (Figures 6(d) and 6(e)). Whether the alpha-cell mass may be dependent on the stage of disease remains to be ascertained. Moreover, it would be interesting to understand when (and possibly to which extent) exercise may represent a strategy to maintain normal islet architecture in diabetes. A further in-depth analysis is obviously required to address this; however, a putative capability of the exercising mice to oppose the diabetic deterioration that progressively sacrifices *β*-cells and damage the pancreatic islet remains intriguing [37]. Still, the mechanisms by which exercise might be responsible for these effects need to be elucidated.

An alternate explanation would be that the selected intensity/amount of exercise for this running study was revealed to be too stressful for this NOD mouse model and therefore inappropriate to test the working hypothesis and meet the soft and hard endpoints [38, 39].

Our explorative studies set the base to further evaluate the implementation of likely exercise protocols along with the dosage of several autoimmunity markers and cytokine and chemokine profiles.

## 5. Conclusions

In NOD mice moderate-intensity exercise seemed to exert glucose-lowering effects only in the late states of diabetes.

Further studies are needed to clarify the utility of the NOD mouse model to mimic and investigate the exercise effects in T1D, immunomodulation, and inflammation. Specifically, dose-response studies in which exercise will be administered to NOD mice at various levels of intensity will be necessary to determine the optimal regimen of physical exercise having clear-cut preventive effects on the development of T1D. A better understanding of the impact of exercise on immune function will be of assistance in designing improved treatments for patients with autoimmune diabetes in the clinical arena.
